# Impact of Albumin Pre-Coating on Gold Nanoparticles Uptake at Single-Cell Level

**DOI:** 10.3390/nano12050749

**Published:** 2022-02-23

**Authors:** Tao Li, Yun Wang, Meng Wang, Lingna Zheng, Wanqin Dai, Chunlei Jiao, Zhuda Song, Yuhui Ma, Yayun Ding, Zhiyong Zhang, Fang Yang, Xiao He

**Affiliations:** 1 Hebei Provincial Key Laboratory of Green Chemical Technology & High Efficient Energy Saving, School of Chemical Engineering and Technology, Hebei University of Technology, Tianjin 300130, China; litao95@ihep.ac.cn; 2 CAS Key Laboratory for Biomedical Effects of Nanomaterials and Nanosafety, CAS-HKU Joint Laboratory of Metallomics on Health & Environment, Institute of High Energy Physics, Chinese Academy of Sciences, Beijing 100049, China; yunwang@ihep.ac.cn (Y.W.); wangmeng@ihep.ac.cn (M.W.); zhengln@ihep.ac.cn (L.Z.); daiwanqin@ihep.ac.cn (W.D.); jiaocl@ihep.ac.cn (C.J.); songzhuda@ihep.ac.cn (Z.S.); mayh@ihep.ac.cn (Y.M.); dingyy@ihep.ac.cn (Y.D.); 3 School of Physical Sciences, University of the Chinese Academy of Sciences, Beijing 100049, China

**Keywords:** gold nanoparticles, bovine serum albumin, sedimentation, agglomeration, cellular uptake

## Abstract

Nanoparticles (NPs) suspension is thermodynamically unstable, agglomeration and sedimentation may occur after introducing NPs into a physiological solution, which in turn affects their recognition and uptake by cells. In this work, rod-like gold NPs (AuNRs) with uniform morphology and size were synthesized to study the impact of bovine serum albumin (BSA) pre-coating on the cellular uptake of AuNRs. A comparison study using horizontal and vertical cell culture configurations was performed to reveal the effect of NPs sedimentation on AuNRs uptake at the single-cell level. Our results demonstrate that the well-dispersed AuNRs-BSA complexes were more stable in culture medium than the pristine AuNRs, and therefore were less taken up by cells. The settled AuNRs agglomerates, although only a small fraction of the total AuNRs, weighed heavily in determining the average AuNRs uptake at the population level. These findings highlight the necessity of applying single-cell quantification analysis in the study of the mechanisms underlying the cellular uptake of NPs.

## 1. Introduction

Nanotechnology provides opportunities to manipulate or develop versatile nanoparticles (NPs) for a wide variety of applications [1,2,3], where conventional techniques may reach their limits. As a result, NPs are inevitably being released into the managed and natural ecosystems. In the past decade, the interaction of NPs with biological systems (nano-bio interaction) has received growing attention, considering the potential health risks following environmental exposure and the rapid development of nanomedicines [4,5,6,7]. When NPs enter a physiological environment, they rapidly adsorb proteins forming what is known as the protein corona. The formation of protein corona alters NPs’ size and interfacial chemistry, affects their dispersion/agglomeration state, and reshapes their biological identity [8]. As a result, the composition and structure of protein corona largely determine the adhesion of NPs to the cell membrane and the subsequent cellular responses, including cellular recognition, uptake, and toxicity. Therefore, understanding the effects of protein corona on nano-bio interactions is of fundamental importance to wide-ranging fields from nanotoxicology to drug delivery [9].

Gold NPs possess many advantages such as facile synthesis, controllable size and morphology, good biocompatibility and chemical stability, and unique optical properties. These features make Gold NPs compatible with a wide range of applications in the biomedical field [10,11,12], and also make them ideal for studying protein-NPs interactions [13,14,15]. The gold NPs synthesized by various optimized methods can achieve high uniformity in size and morphology, and can remain chemical stable during the interaction with proteins, therefore reducing variables in the study. They have strong surface plasmon resonance (SPR) absorbance, and the shift or broadening of SPR peak are often regarded as signs of NPs agglomeration [16,17]. Therefore, gold NPs are frequently used to investigate the formation of protein corona on NPs surface as well as its biological consequences [18,19,20,21]. The formation and dynamic evolution of protein corona can significantly affect the immune recognition and uptake of gold NPs by cells, involving processes and factors such as immunoglobulin deposition followed by complement opsonization [22], structure and composition of the protein layer [23,24], the steric hindrance of inner layer proteins by outer layer proteins [25], the agglomeration of gold NPs [26], etc. To date, our knowledge of the effects of protein corona on NPs uptake has been largely derived from the overall exposure-response metric, with a lack of understanding at the single-cell level.

However, recent advancements in single-cell analysis clearly show heterogeneity in cell populations previously assumed to be identical [27,28]. The heterogeneity mainly stems from the non-uniform properties of NPs themselves and the complexity of the biological microenvironment. The formation of protein corona will further introduce new variables to the interaction between cells and NPs. Therefore, there is an emerging consensus that experimental methods that provide information about average population-level cellular responses to NPs are insufficient, and sometimes potentially misleading [29]. In this work, rod-like gold NPs (AuNRs) with uniform morphology and size were synthesized to study how the pre-coating of AuNRs with bovine serum albumin (BSA) would affect the dispersion of AuNRs in culture medium, and the subsequent cellular uptake of AuNRs. AuNR uptake at the single-cell level was determined by single-cell ICP-MS (SC-ICP-MS) equipped with a high-efficiency cell introduction system as described previously [30]. Similar to a previous study [31], we investigated the effect of NPs sedimentation on cellular NPs uptake by exposing cells cultured in horizontal and vertical configurations to AuNRs.

## 2. Materials and Methods

### 2.1. Materials

Sodium borohydride (NaBH_4_), ascorbic acid (AA), tetrachloroaurate acid (HAuCl_4_∙4H_2_O), hexadecyl trimethyl ammonium bromide (CTAB), sodium oleate (NaOL), and BSA were purchased from Sigma (≥98%, Darmstadt, Germany). Mouse RAW264.7 cells were cultured in Dulbecco modified Eagle’s medium (DMEM, Hyclone, Logan, UT, USA) supplemented with 10% fetal bovine serum (FBS, Gibco, Carlsbad, CA, USA) and 1% antibiotic-mycotic (penicillin-streptomycin, 10,000 U/mL, Gibco, Carlsbad, CA, USA). Ultra-pure water was prepared by Milli-Q ultra-pure water system (18.2 MΩ/cm).

### 2.2. Preparation of AuNRs

A typical seed-mediated growth method was used to synthesize AuNRs as described previously [32]. Briefly, the seed solution was made by mixing 5 mL of HAuCl_4_ (0.5 mM), 5 mL of CTAB (0.2 M), and 0.6 mL of fresh ice-cold NaBH_4_ (0.01 M) under vigorous stirring. The growth solution consisted of a mixture of 1.4 g CTAB and 0.2468 g NaOL in 50 mL of warm water (~50 °C) in a 250 mL Erlenmeyer flask. After cooling down the solution to 30 °C, 3.6 mL of 4 mM AgNO_3_ and 50 mL of 1 mM HAuCl_4_ were mixed for a static duration of 15 min, and then stirred at 700 rpm for 90 min. After another 15 min of gentle stirring, 0.25 mL of ascorbic acid (0.064 M) was added under vigorously stirring followed by the addition of 80 μL of seed solution. The resultant was stirred for 30 s and left standing at 30 °C overnight. The final products were isolated by centrifugation at 10,000 g for 20 min and washed with ultrapure water 3 times before characterization. The size and morphology of AuNRs were observed by transmission electron microscopy (TEM, Hitachi HT7700, Tokyo, Japan) and scanning electron microscopy (SEM, Hitachi S4800, Tokyo, Japan). Their hydrodynamic distribution and surface charge were determined using dynamic light scattering (DLS, Zetasizer ZS90, Malvern, UK). Optical extinction spectra were recorded using a UV-Vis spectrophotometer (Shimadzu UV2700, Suzhou, China).

### 2.3. Pre-Coating AuNRs with BSA

The suspension of AuNRs was diluted to 0.2 mg/mL and ultrasonicated for 15 min, then incubated on standing with BSA at concentration ratios of 10 and 100 (BSA:AuNRs) at 25 °C for 12 h. BCA method was used to determine the amount of BSA adsorbed by AuNRs. The agglomeration and sedimentation of AuNRs@BSA_10_ and AuNRs@BSA_100_ were characterized with TEM, SEM, DLS, and UV-Vis assays. After the pre-incubation, the BSA-AuNRs complexes (termed as AuNRs@BSA_10_ and AuNRs@BSA_100_) were added in the culture medium.

### 2.4. Cell Culture

RAW264.7 cells were seeded on polystyrene substrates with an initial density of 10^6^ cells/mL in a humidified atmosphere with 5% CO_2_ at 37 °C. When cells reached 80% confluence, half of the substrates were changed from horizontal to vertical, and all substrates were immersed in the refreshed culture medium containing 2 μg/mL of AuNRs, AuNRs@BSA_10_, or AuNRs@BSA_100_, respectively. After a 2-h exposure, cells cultured horizontally to vertically were harvested separately and washed several times. Cells were resuspended, fixed with 2.5% glutaraldehyde solution, and counted with a cell counter. Then, the uptake of AuNRs was determined with ICP-MS (Thermo X7, Waltham, MA, USA) after aqua regia digestion, or directly with SC-ICP-MS (PerkinElmer NexION 300D, Shelton, CT, USA) [30].

## 3. Results

### 3.1. Characterization of AuNRs

**Figure 1:**
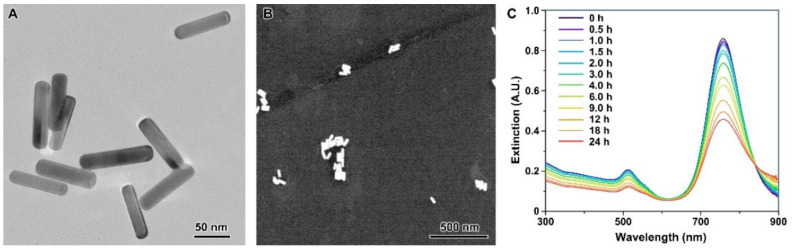
TEM (**A**) and SEM (**B**) images of AuNRs. (**C**): the extinction spectra of 10 μg/mL AuNRs in ultrapure water within a 24-h standing.

As shown in Figure 1, the AuNRs synthesized in this work were uniform in size and morphology, with a mean length of 84.7 ± 1.3 nm, and a mean width of 22.2 ± 0.6 nm (Figure 1A). DLS assay shows that the hydrodynamic diameter of AuNRs in ultrapure water was 80.3 ± 37.1 nm, and the zeta-potential was 19.7 ± 6.9 mV. The result shows that the AuNRs had good dispersibility in ultrapure water, though agglomeration of AuNRs could still be found more or less under SEM (Figure 1B). UV-Vis spectroscopy shows a time-dependent decline in extinction, and no peak (the maximum extinction) red-shifts were observed (Figure 1C). However, the extinction above 850 nm increased with time. These results suggest that the spectral changes were mainly caused by AuNRs sedimentation, and meanwhile a small fraction of the AuNRs were agglomerated.

### 3.2. AuNRs Agglomeration and Sedimentation after BSA Pre-Coating

**Figure 2:**
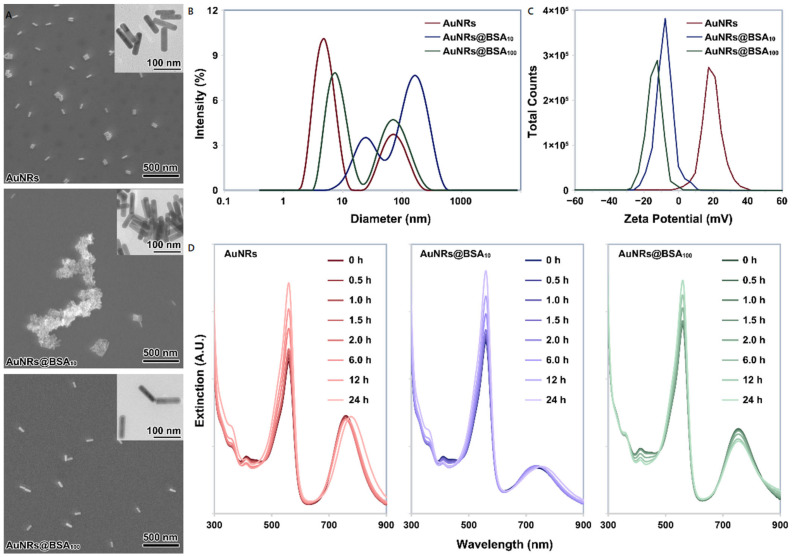
(**A**): TEM and SEM images of AuNRs, AuNRs@BSA_10_ and AuNRs@BSA_100_, respectively; (**B**,**C**): the hydrodynamic distribution and zeta-potential of AuNRs, AuNRs@BSA_10_ and AuNRs@BSA_100_ resuspended in ultrapure water determined by DLS; (**D**): the extinction spectra of AuNRs, AuNRs@BSA_10_ and AuNRs@BSA_100_ dispersed in DMEM culture medium supplemented with 10% FBS and 1% antibiotic-mycotic.

In the present work, AuNRs were pre-incubated with BSA at concentration ratios of 10 and 100 (BSA:AuNRs) for 12 h. Our results demonstrated that protein corona did not always aid dispersion and stabilization of NPs, instead, the incubation of 0.2 mg/mL AuNRs with 2.0 mg/mL BSA resulted in severe agglomeration of AuNRs (Figure 2A,B). The enhanced agglomeration at low protein-to-NP ratios could be attributed to two possibilities: (i) negatively charged proteins neutralized the positive surface of AuNRs, resulting in weakened colloidal stability depending on electrostatic repulsion (Figure 2C), and (ii) unfolded BSA-BSA interactions triggered the agglomeration process of AuNRs [33]. It should be noticed that despite the presence of large-sized agglomerates, there were still a few AuNRs@BSA_10_ that remained monodisperse (Figure 2A). When incubating AuNRs with BSA at a high protein-to-NP ratio, the solubilizing function of BSA dominates due to a complete surface coverage. Correspondingly, AuNRs@BSA_100_ had a more constant extinction (Supplementary Materials Figure S1) than AuNRs (Figure 1C) within 24 h. SEM and TEM images also show that most of the AuNRs@BSA_100_ kept monodisperse, while some of the AuNRs agglomerated slightly. But DLS results suggested that AuNRs and AuNRs@BSA_100_ had similar hydrodynamic diameters (Figure 2B), which may be due to DLS result describes only the NPs and their small agglomerates in suspensions while larger agglomerates have settled already.

Agglomeration of NPs during measurements in relevant biological media is a frequent problem in NPs characterization [34]. In this work, AuNRs, as well as their complexes with BSA, were introduced into the culture medium to a final concentration of 10 μg/mL, where they interacted with 10% FBS at an even higher protein-to-NP ratio (~300). Due to the presence of abundant salts and proteins, SEM and DLS failed to measure the size distribution of AuNRs in the culture medium, although the appearance of the suspension suggested improved dispersion of AuNRs. Since previous work has tried to correlate the SPR absorbance spectrum of gold NPs and the size distribution of agglomerates [34], the optical extinction spectra of the culture media containing AuNRs, AuNRs@BSA_10_ and AuNRs@BSA_100_ were recorded (Figure 2D). The changes in peak at 560 nm were mainly caused by phenol red in the medium (Figure S2), so this study just focuses on the changes of the peak around 750 nm. Within the first 2 h of mixing AuNRs or AuNRs-BSA complexes with the medium, there were slight drops in maximum extinction around 750 nm for all 3 treatments (too subtle to show clearly). But when we extended the observation to 24 h, red-shifts and slight recoveries in height of SPR peak could be found for both AuNRs and AuNRs@BSA_10_. The red-shifts of the SPR band can be ascribed to the increasing agglomerate sizes across the whole size distribution [16,34], or the change of the refractive index at the vicinity of AuNRs [35], resulting from the dynamic NP-protein interaction. The recovery of the maximum extinction also implies that a small amount of settled agglomerates were suspended again or some large-sized agglomerates were deagglomerated into smaller ones. The SPR peak of AuNRs@BSA_100_ kept stable at the beginning, with only a small decrease in the middle and late stages of the incubation. This may be because the inner layer of protein corona on the surface of AuNRs@BSA_100_ had hardened [35], thus minimizing the dynamic exchange of inner layer proteins with the surrounding solution. Extinction above 850 nm increased with time, suggesting the appearance of AuNRs@BSA_100_ agglomeration. These results together suggest that AuNRs and their complexes with BSA had different sedimentation and diffusion in the culture medium. In the following, we therefore investigated how BSA pre-coating affects the AuNRs uptake.

### 3.3. Cellular Uptake of AuNRs

Previous work used upright and inverted cell culture configurations to demonstrate the effect of sedimentation and diffusion on the cellular uptake of gold NPs [31]. Similarly, we investigated the AuNRs uptake across cell populations by exposing cells cultured in horizontal and vertical configurations to AuNRs at a concentration of 2 μg/mL. To minimize the influence of cell deformation under gravity on the exposed area and uptake activity of the cells cultured vertically, we only measured the cellular uptake of AuNRs within 2 h.

**Figure 3:**
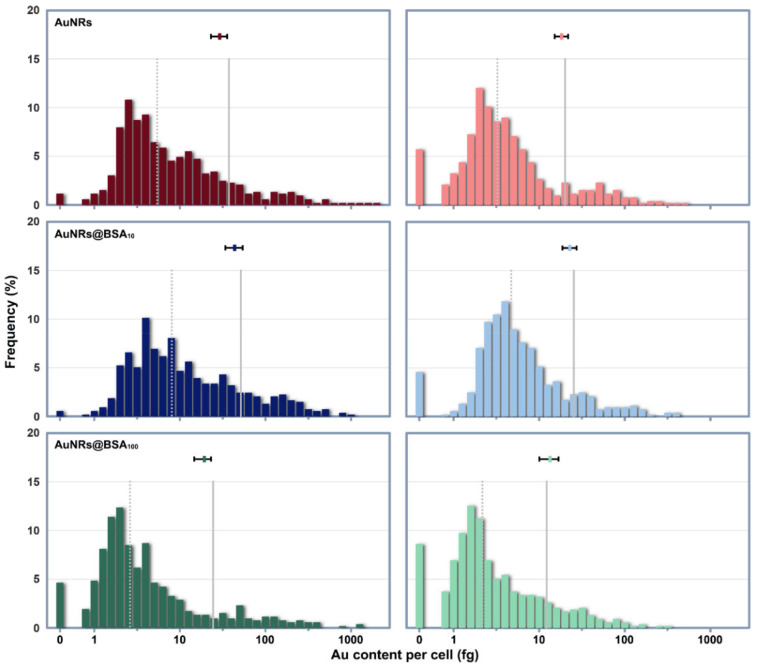
Histogram shows the log-normal distribution of Au mass across cell populations cultured horizontally (**Left**) or vertically (**Right**). The grey, vertical dotted and solid lines represent the median and mean of Au mass across cell populations respectively. The mean and SD of Au mass per cell of each treatment determined from cell digestion analysis with solution ICP-MS are presented by plot and horizontal error bars in each panel.

Results from both the digested cell populations and the cells as individuals indicate that BSA pre-coating would significantly affect the cellular uptake of AuNRs (Figure 3). Cell digestion analysis showed that the average AuNRs uptakes in horizontally cultured cells increased in the following order: AuNRs@BSA_100_ < AuNRs < AuNRs@BSA_10_ (*p* < 0.05 among the treatments). When cultured vertically, a significant difference was only found between AuNRs@BSA_100_ and AuNRs@BSA_10_ treatments (*p* < 0.05). Cells cultured horizontally took up more AuNRs and AuNRs@BSA_10_ than those cultured vertically (*p* < 0.05), suggesting that the sedimentation of AuNRs agglomerates had an impact on AuNRs uptake by cells. AuNRs@BSA_100_ were dispersed well in the culture medium, therefore cellular uptake was not significantly affected by the culture configurations. Single-cell quantification indicates that Au mass in exposed cell populations was heterogeneous and spanned over approximately 2 orders of magnitude (Figure 3). After a 2-h exposure, Au contents in most cells were between 1–10 fg, but cells cultured horizontally had a higher probability of taking up more than 100 fg of AuNRs than cells cultured vertically.

## 4. Discussion

Cellular uptake of NPs in vitro is typically measured by exposing cells at the bottom of a culture plate to NPs suspensions, and it is generally assumed that the NPs are well-dispersed. However, NPs are present in the culture medium as thermodynamically unstable suspensions and tend to agglomerate or be encapsulated by proteins, resulting from the necessity of NPs to minimize their surface energy. Severe agglomeration may lead to the sedimentation of NPs, while the formation of protein corona is generally considered to improve the dispersity of NPs [35,36]. When AuNRs were introduced into the culture medium, two opposing kinetic processes were competing at the same time: (i) the destabilization of AuNRs promoted by the high ionic strength and (ii) the stabilization of AuNRs against agglomeration via protein adsorption. If the protein corona can form prior to the introduction of AuNRs into the medium with high ionic strength, then the resultant colloidal solution will remain stable.

However, our findings suggest that although the abundant proteins in the culture medium could keep most AuNRs dispersed in vitro, the agglomeration and sedimentation of a few NPs are inevitable. The comparison study using different cell culture configurations can clearly present the effects of NPs sedimentation on the cellular uptake of AuNRs. We found that the long tail at the high end of the AuNRs uptake distribution had a great influence on the average Au mass in horizontally cultured cells. The settled AuNRs agglomerates could directly contact the cell surface, therefore had a greater weight in determining the average Au mass per cell, though they constituted only a small fraction of all AuNRs. Relatively, those suspended NPs (as well as their agglomerates) are less weighted in determining cellular uptake when compared with the settled agglomerates. When cultured vertically, fewer cells appeared at the high end of the distribution of intracellular Au mass. About half of the vertically cultured cells exposed to AuNRs@BSA_100_ took up no more than 2.2 fg of Au. Such a high incidence of low Au uptake may not be solely because more AuNRs@BSA_100_ were monodisperse, but also because BSA pre-coating depressed the incidence of cellular recognition and uptake of AuNRs. Therefore, our single-cell quantification confirmed the stealth effect of BSA coating on NPs, consistent with previous reports [37,38]. But nevertheless, cellular uptake of AuNRs in vertically cultured cells was still quite heterogeneous. These results also demonstrate that the limitation of determining NPs uptake from large population averages leads to potentially misleading information and the lack of any information about cellular heterogeneity.

Our findings suggest that the heterogeneity in cellular uptake of AuNRs is an indication of the heterogeneous distribution of AuNRs in the culture medium. We have to keep in mind that NPs do not form solutions, but colloidal dispersions, which are multi-phase systems and thermodynamically unstable [39]. Even if the macroscopic nature of NPs suspension remains stable, their agglomeration and size distribution are constantly changing. Resulting from diffusion and sedimentation, large-sized NPs agglomerates have a certain probability to reach the cell surface. Their large surface and the large number of proteins adsorbed on their surface, make them more likely than small agglomerates and monodisperse NPs to activate receptor-mediated cellular recognition and internalization. Therefore, this work provides evidence at the single-cell level that the cellular NPs mass distribution is driven by dynamic processes rather than by equilibrium partitioning [40]. Furthermore, this work also brings some considerations regarding the experimental design and dosimetry of NPs exposure. For example, the reason that some surface-modified NPs are less taken up by cells than their unmodified counterparts may sometimes simply be (at least in part) that the surface modification improves the dispersibility of the NPs and thus reduces the incidence of cellular uptake of large-sized agglomerates in horizontally cultured cells. Therefore, it is necessary to use more single-cell quantification analysis in studies of the sedimentation and cellular uptake of NPs, and to consider the influence of cell culture configuration.

## 5. Conclusions

This work studied the impact of BSA pre-coating on the sedimentation and cellular uptake of AuNRs. Our results demonstrate that BSA pre-coating at a high BSA-to-AuNRs ratio (100:1) could well facilitate the dispersion of AuNRs while a low BSA-to-AuNRs ratio (10:1) resulted in severe agglomeration and sedimentation of AuNRs. The well-dispersed AuNRs-BSA complexes were more stable in culture medium than pristine AuNRs, and thus leading to a lower average Au mass per cell by reducing the formation and sedimentation of large-sized agglomerates of AuNRs. The settled AuNRs agglomerates, although only a small fraction of the total AuNRs, carry great weight in determining the average AuNRs uptake at the population level. NPs quantification at the single-cell level can reveal the heterogeneity in cellular uptake of NPs, thereby more granularly reflecting the impact of protein corona formation. This study therefore highlights the necessity of applying single-cell quantification techniques in the study of the mechanisms underlying cellular uptake of NPs.

## Data Availability

Not applicable.

## Supplementary Materials

The following supporting information can be downloaded at: https://www.mdpi.com/article/10.3390/nano12050749/s1, Figure S1: The extinction spectra of 10 μg/mL AuNRs@BSA_100_ in ultrapure water within a 24-h standing.; Figure S2: The extinction spectra of cell culture medium within a 24-h standing.
