# Supplementary material for: Impact of Albumin Pre-Coating on Gold Nanoparticles Uptake at Single-Cell Level
Source: Nanomaterials (Basel). 2022 Feb 23;12(5):749. doi: 10.3390/nano12050749 (PMC8911762; doi:10.3390/nano12050749)
Supplement: Supplementary file 1 [file nanomaterials-12-00749-s001.zip › nanomaterials-1609480-supplementary.pdf]

Supporting Information

Impact of Albumin Pre-Coating on Gold Nanoparticles Uptake at Single-Cell Level

Tao Li [1], Yun Wang [2,3], Meng Wang [2], Lingna Zheng [2], Wanqin Dai [2,3], Chunlei Jiao [2,3], Zhuda Song [2,3], Yuhui Ma [2], Yayun Ding [2], Zhiyong Zhang [2,3,*], Fang Yang [1,*] and Xiao He [2,*]

1 Hebei Provincial Key Lab of Green Chemical Technology & High Efficient Energy Saving, School of Chemical Engineering and Technology, Hebei University of Technology, Tianjin 300130, China; litao95@ihep.ac.cn

2 CAS Key Lab for Biomedical Effects of Nanomaterials and Nanosafety, CAS-HKU Joint Laboratory of Metallomics on Health & Environment, Institute of High Energy Physics, Chinese Academy of Sciences, Beijing 100049, China; yunwang@ihep.ac.cn (Y.W.); wangmeng@ihep.ac.cn (M.W.); zhengln@ihep.ac.cn (L.Z.); daiwanqin@ihep.ac.cn (W.D.); jiaocl@ihep.ac.cn (C.J.); songzhuda@ihep.ac.cn (Z.S.); mayh@ihep.ac.cn (Y.M.); dingyy@ihep.ac.cn (Y.D.)

3 School of Physical Sciences, University of the Chinese Academy of Sciences, Beijing 100049, China

* Correspondence: zhangzhy@ihep.ac.cn (Z.Z.); yangfang@hebut.edu.cn (F.Y.); hexiao@ihep.ac.cn (X.H.)

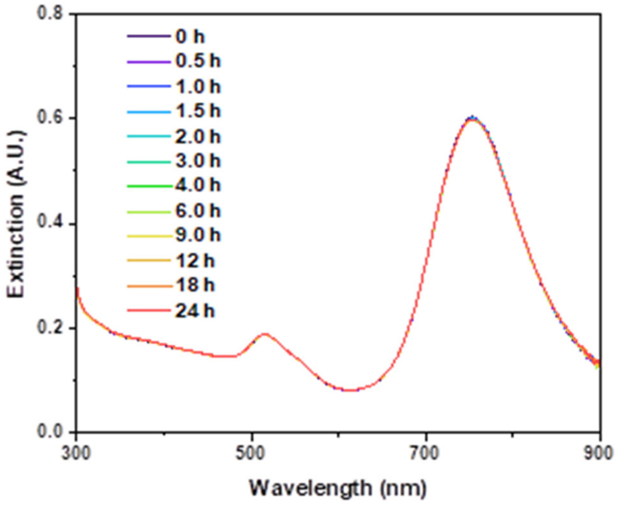

Figure S1. The extinction spectra of 10 μg/mL AuNRs@BSA_{100} in ultrapure water within a 24-h standing.

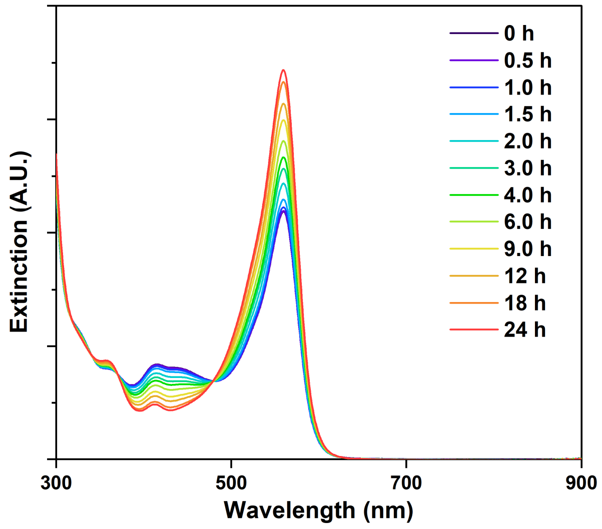

Figure S2. The extinction spectra of cell culture medium within a 24-h standing.
